# Viruses Infecting the Plant Pathogenic Fungus *Rhizoctonia solani*

**DOI:** 10.3390/v11121113

**Published:** 2019-11-30

**Authors:** Assane Hamidou Abdoulaye, Mohamed Frahat Foda, Ioly Kotta-Loizou

**Affiliations:** 1 State Key Laboratory of Agricultural Microbiology, College of Plant Science and Technology, Huazhong Agricultural University, Wuhan 430070, China; doul_as@yahoo.fr; 2 State Key Laboratory of Agricultural Microbiology, College of Veterinary Medicine, Huazhong Agricultural University, Wuhan 430070, China; 3 Department of Biochemistry, Faculty of Agriculture, Benha University, Moshtohor, Toukh 13736, Egypt; 4 Department of Life Sciences, Imperial College London, London SW7 2AZ, UK; i.kotta-loizou13@imperial.ac.uk

**Keywords:** *Rhizoctonia solani*, mycovirus, (+)/(−)ssRNA, dsRNA, hyper/hypovirulence, virus–host interactions

## Abstract

The cosmopolitan fungus *Rhizoctonia solani* has a wide host range and is the causal agent of numerous crop diseases, leading to significant economic losses. To date, no cultivars showing complete resistance to *R. solani* have been identified and it is imperative to develop a strategy to control the spread of the disease. Fungal viruses, or mycoviruses, are widespread in all major groups of fungi and next-generation sequencing (NGS) is currently the most efficient approach for their identification. An increasing number of novel mycoviruses are being reported, including double-stranded (ds) RNA, circular single-stranded (ss) DNA, negative sense (−)ssRNA, and positive sense (+)ssRNA viruses. The majority of mycovirus infections are cryptic with no obvious symptoms on the hosts; however, some mycoviruses may alter fungal host pathogenicity resulting in hypervirulence or hypovirulence and are therefore potential biological control agents that could be used to combat fungal diseases. *R. solani* harbors a range of dsRNA and ssRNA viruses, either belonging to established families, such as *Endornaviridae*, *Tymoviridae*, *Partitiviridae*, and *Narnaviridae*, or unclassified, and some of them have been associated with hypervirulence or hypovirulence. Here we discuss in depth the molecular features of known viruses infecting *R. solani* and their potential as biological control agents.

## 1. Introduction

The genus *Rhizoctonia* was initially described by French mycologist Augustin Pyramus de Candolle in 1815 [1] and belongs to the order Cantharellales, phylum Basidiomycota. *Rhizoctonia* species are assigned into three main groups based on the number of nuclei in the fungal cells: Uninucleate *Rhizoctonia*, binucleate *Rhizoctonia* (teleomorphs: *Ceratobasidium* spp. and *Tulasnella* spp.) and multinucleate *Rhizoctonia* (teleomorphs: *Thanatephorus* spp. and *Waitea* spp.). *Rhizoctonia solani* Kühn (teleomorph: *Thanatephorus cucumeris*) is the most widely known species within the group of multinucleate *Rhizoctonia* and is classified into fourteen anastomosis groups (AGs) based on hyphal fusion experiments (Table 1) [2,3,4,5,6]. *R. solani* is a soil-borne plant pathogen with widespread geographical distribution and a wide host range, known to cause various important crop diseases, leading to significant agricultural and economic losses. For instance, *R. solani* is the causative agent of rice sheath blight leading up to 50% yield losses in Asia [4]. The symptoms caused by *R. solani* infection vary depending on the host plant and include damping-off of seedlings, stem canker, and root or stem rots [7].

The establishment of *R. solani* infection in a suitable host occurs following the attachment of fungal mycelia or sclerotia on the host root. A sclerotium is an aggregate of a dense structure of clustered mycelium with the ability to overwinter several years in host plant tissue, plant debris or soil and germinate in the presence of root exudates emitted by the plant when climatic condition are favorable [5]. The fungus then proliferates on the root and produces specialised T-shaped structures named “infection pads”. These infection pads produce enzymes capable of digesting the plant cell wall so that the fungus penetrates and colonizes the intercellular and intracellular spaces of the root tissue. As it develops, the fungus diverts the cellular reserves of the plant for its own growth. Gradually, the mycelium invades the cells and kills them, while producing survival structures, and the plant begins to wither when its conducting vessels are attacked [5,8].

Attempts to control *R. solani* by agronomic approaches, such as breeding strategies, crop rotation or chemical fungicides, proved ineffective due to the wide host range, soil-borne nature and the saprotrophitic nature of the fungus. Even in cases of extensive use of chemical fungicides due to substantial crop losses, *R. solani* proved persistent. Notably, *R. solani* does not produce conidia (asexual spores), therefore its ability to spread long distances is limited, despite being considered ubiquitous in soil [9,10]. Therefore, it is imperative to find new alternatives, preferably with minimal impact on the environment, to protect crops from *R. solani* while reducing the use of chemical fungicides [11]. Moreover, it is known that *R. solani* hosts a range of viruses, some of them still unclassified [7,12,13,14,15,16,17,18,19,20,21,22].

A virus is an infectious agent requiring a host, such as a cell, whose metabolism and constituents it uses to replicate. A mycovirus is a virus that specifically infects fungi. The first mycovirus was found in the edible mushroom *Agaricus bisporus* (phylum: Basidiomycota) in 1962 [23]. Since then mycoviruses have been found in all major fungal taxa, namely Basidiomycota, Ascomycota, Chytridiomycota, Deuteromycota, and Zygomycota [13]. However, it is considered that only a fraction of the extant mycoviruses have been described so far and next-generation sequencing (NGS) techniques are currently being used to identify novel unknown mycoviruses [9]. The majority of mycoviruses reported have dsRNA genomes, although ssRNA and DNA viruses have been reported [13]. According to the International Committee for the Taxonomy of Viruses (ICTV; 2018b), mycoviruses are currently classified in nineteen officially recognized families and a floating genus not classified in a family, accommodating linear dsRNA viruses (*Amalgaviridae*, *Botybirnavirus, Chrysoviridae*, *Megabirnaviridae*, *Partitiviridae*, *Quadriviridae*, *Reoviridae*, *Totiviridae*), linear positive-sense (+)ssRNA families (*Alphaflexiviridae*, *Barnaviridae*, *Botourmiaviridae, Deltaflexiviridae, Endornaviridae, Gammaflexiviridae*, *Hypoviridae, Narnaviridae*), reverse transcribing linear ssRNA families (*Metaviridae*, *Pseudoviridae*), linear negative-sense (−)ssRNA families (*Mymonaviridae*) and circular ssDNA viruses (*Genomoviridae*) [24,25]. Generally, mycoviruses lack an extracellular phase in their replication cycle [9,23]; nevertheless, a novel ssDNA virus related to plant geminiviruses and conferring hypovirulence to its host, is transmitted in aerosols [26]. Fungi infected by viruses often present unusual characteristics such as abnormal pigmentation, irregular growth, and altered sexual reproduction. Potential hypovirulent effects of mycoviruses on their fungal hosts may be used for biological control of fungal diseases, similar to the application of a hypovirus found in the plant pathogenic fungus *Cryphonectria parasitica* used to control chestnut blight in Europe [12].

## 2. The Diversity of Viruses Infecting *Rhizoctonia solani*

**Figure 1:**
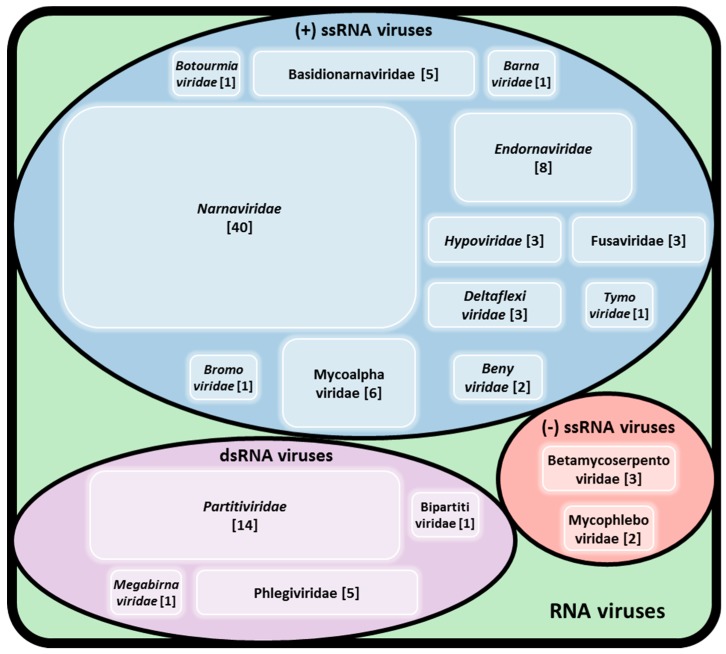
Virus families reported infecting *R. solani*. The numbers in brackets refer to the number of different viruses belonging to each family and reported to infect *R. solani*.

The first dsRNA element in *R. solani* was initially described by Butler and Castano [13]. Since then numerous thorough studies were performed to explore the diversity of viruses infecting *R. solani*. To date, approximately 100 viruses have been found in *R. solani* isolates, including members of established families accommodating dsRNA, (+)ssRNA, and (−)ssRNA together with members of proposed families and unclassified RNA elements (Figure 1). Some of the viruses reported to infect *R. solani* belong to well-studied mycovirus families, such as *Barnaviridae*, *Botourmiaviridae*, *Deltaflexiviridae, Endornaviridae*, *Hypoviridae*, *Megabirnaviridae*, *Narnaviridae*, and *Partitiviridae* (Table 2 and Table S1). Others belong or are closely related to families traditionally known to infect plants, such as CMV [7] and proposed members of the orders *Bunyavirales*, *Serpentovirales*, and *Tymovirales* [6,27,28,29].

In general, recent large-scale metatranscriptomic analyses of plant pathogenic fungi has led to the discovery of several (−)ssRNA mycoviruses including mymonaviruses [19,30,31,32] and other mycoviruses related to the bi- and tri-segmented peribunyaviruses and phenuiviruses, and the multi-segmented ophioviruses [19,33,34]. Since whole transcriptome shotgun sequencing is widely utilized to identify and quantify mycoviruses in biological samples, the majority of reported sequences are partial while verification of full length genomic sequences is not always feasible. To our knowledge, the mycoviruses infecting *R. solani* whose complete genome sequences have been reported so far belong to the established families *Deltaflexiviridae*, *Endornavirdae*, and *Partitiviridae* [15] but several partial genomes of viruses have been described (Table 2 and Table S1) [6,19]. Only the betapartitivirus Rhizoctonia solani virus 717 and the magoulivirus Rhizoctonia solani ourmia-like virus have been approved by ICTV so far, even though the genome of the latter has not been fully sequenced.

To date, numerous different viruses have been reported to infect *R. solani* AG-1 IA, isolated from rice: Rhizoctonia solani dsRNA virus 1 (RsRV1) in 2013 [16], Rhizoctonia solani partitivirus 2 (RsPV2) in 2014 [17], Rhizoctonia solani RNA virus 2 (RsRV2-HN008) in 2015 [12] and more recently Rhizoctonia solani dsRNA virus 3 (RsRV3) [35], Rhizoctonia solani partitivirus 3 to 8 (RsPV3 to 8, respectively) [36,37,38] and Rhizoctonia solani endornavirus 1 (RsEV1) [39]. Multiple co-infections of *R. solani* isolates are not uncommon; for instance *R. solani* AG2-2 IV DC17 has been reported to harbor an endornavirus, a megabirnavirus, a mitovirus, two flexiviruses, and three closely related mycoalphaviruses [9,15]. Similarly, *R. solani* AG-3PT RS002 infecting potato harbors an endornavirus [14] and a mitovirus [18]. Furthermore, a cross-kingdom viral infection has been discovered when a plant virus, cucumber mosaic virus (CMV), was found in an *R. solani* strain isolated from potato plants [7]. In addition, five unrelated dsRNA elements (L1, L2, M1, M2, and S1) were found to occur in DNA form in *R. solani* AG3 from North America [40,41].

### 2.1. Double-Stranded RNA Viruses

DsRNA viruses have a wide host range including animals, plants, protozoa, and fungi [42]. Mycoviruses with dsRNA genomes are mostly encapsidated in isometric particles [43] and are currently classified into eight families: *Amalgaviridae* (1 genomic segment, 3.5 kbp in length), *Chrysoviridae* (3–7 genomic segments, 2.4–3.6 kbp in length), *Megabirnaviridae* (2 genomic segments, 7.0–9.0 kbp in length), *Partitiviridae* (2–3 genomic segments, 1.4–2.3 kbp in length), *Quadriviridae* (4 genomic segments, 3.7–4.9 kbp in length), *Reoviridae* (*Spinareovirinae* subfamily, 10–12 genomic segments, 0.7–5.0 kbp in length) and *Totiviridae* (non-segmented, 4.6–7 kbp in length) [42,44,45,46]. Moreover, a dsRNA virus named Botrytis porri RNA virus 1 (BpRV1) belonging to the genus *Botybirnavirus* has been described [47]. Mycovirus taxonomy regularly changes with the discovery of novel viruses [48] and additional families, such as Alternaviridae (4 genomic segments 1.4–3.6 kbp in length), have been proposed recently. Generally, dsRNA viruses form spherical and not filamentous virions, the latter being a common characteristic of several (+)ssRNA plant and fungal viruses including *Alphaflexiviridae*, *Betaflexiviridae*, *Gammaflexiviridae*, *Potyviridae*, and *Closteroviridae*; nevertheless, a novel dsRNA virus from *Colletotrichum camelliae* isolated from tea plants in China was found to form flexuous and elongated virions [42,44]. Some dsRNA viruses form no true virions but are associated with and coated by viral proteins, as reported recently for Aspergillus fumigatus tetramycovirus-1 (AfuTmV-1) and Beauveria bassiana polymycovirus-1 (BbPmV-1), from the human pathogen *A. fumigatus* and the insect pathogen *B. bassiana*, respectively [49,50]. BbPmV-1 appears to be associated with hypovirulence in its host which is uncommon for mycoviruses [51].

#### 2.1.1. *Megabirnaviridae* and Phlegiviridae

*Megabirnaviridae* is a family known to infect fungi and currently accommodates one genus *Megabirnavirus* [52], while a second related genus Phlegivirus has been proposed [9]. Members of the family contain linear bi-segmented dsRNA genomes, with two linear segments sizing each from 7 to 8.9 kbp and comprising 16.1 kbp in total length. The dsRNAs genomes are separately packaged into isometric particles [43,53,54]. The exemplar of the only officially recognized species RnMV1/W779 for each segment two tandem non-overlapping ORFs in each segment [52]. The ORFs in the largest segment encode a putative RdRp and a capsid protein (CP), whereas the ORFs in the smallest segment encode two proteins of unknown function [53,54]. Other unclassified members of the family include Sclerotinia sclerotiorum megabirnavirus 1 (SsMBV1) [55], Rosellinia necatrix megabirnavirus 2 (RnMBV2) [56], Pleospora megabirnavirus 1 (PMBV1) [57], and Entoleuca megabirnavirus 1 (EnMBV1) [27]. Virus transmission occurs either horizontally through anastomosis or vertically via sporulation [53]. Additionally, recent NGS approaches revealed more dsRNA viruses related to the *Megabirnaviridae* family (Table S1) [6,9]. The partial sequence of Rhizoctonia solani megabirnavirus 1 (RsMBV1; Table 2) is 975 bp in length and encodes a putative RdRp (Pfam02123, E-value 7e-13). More partial ORFs have been reported and named Rhizoctonia solani dsRNA virus 6-10 (RsRV6-10; Table S1), which are related to the previously proposed genus Phlegivirus [58] in the family Phlegiviridae [6].

#### 2.1.2. *Partitiviridae* and Bipartitiviridae

Members of the *Partitiviridae* family have two linear, individually encapsidated monocistronic dsRNA segments, while an additional satellite or defective dsRNA segment may also be present. Each dsRNA segment is from 1.4 to 2.4 kbp in size and contains one large ORF encoding and RdRp or CP [59]. The family accommodates five genera: *Alphapartitivirus*, *Betapartitivirus*, *Cryspovirus, Deltapartitivirus*, and *Gammapartitivirus* [59]. The genera *Alphapartitivirus* and *Betapartitivirus* are known to infect plants, ascomycetes or basidiomycetes, whereas the genus *Gammapartitivirus* infect ascomycetes [59] and oomycetes [60]. The genera *Deltapartitivirus* and *Cryspovirus* infect exclusively plants and protozoa, respectively [59]. Fungal partitiviruses are transmitted either horizontally via hyphal fusion or vertically via spores [61].

**Figure 2:**
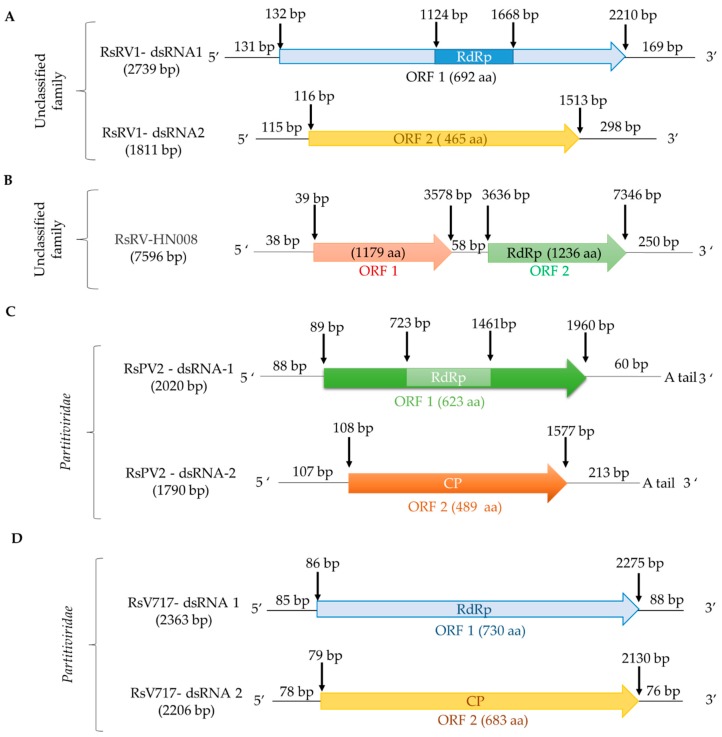
Schematic representation of the genomic organization of double-stranded (ds)RNA viruses infecting *R. solani*: (**A**) unclassified RsRV1/B275; (**B**) unclassified RsRV-HN008/HN008; (**C**) betapartitivirus RsV717/Rhs 717; (**D**) alphapartitivirus RsPV2/GD-11.

To date, members of the genera *Alphapartitivirus* and *Betapartitivirus* have been found in *R. solani*. a putative alphapartitivirus named Rhizoctonia solani partitivirus 2 (RsPV2) was isolated from the causal agent of rice sheath blight, *R. solani* AG-1 IA GD-11. RsPV2/GD-11 contains two segments 2020 bp and 1790 bp in length, respectively (Figure 2; Table 2). The protein encoded by dsRNA1 is an RdRp (Pfam02123, E-value 5e-05) similar to that of partitiviruses such as Diuris peduncolata cryptic virus (DpCV; accession number JX156424, identity 63.77%, E-value 0.0), while dsRNA2 encodes a CP [17]. The betapartitivirus Rhizoctonia solani virus 717 (RsV717), isolated from *R. solani* AG-2 Rhs 717 has two genomic segments, 2363 bp and 2206 bp in length (Figure 2; Table 2). DsRNA1 encodes a putative RdRp (Pfam00680, E-value 0.002) with high similarity to that of Fusarium poae virus 1 (FpV1; accession number LC150606, identity 46.81%, E-value 0.0); while dsRNA2 encodes a putative CP [20]. In addition, the complete genomes of four other alphapartitiviruses, Rhizoctonia solani dsRNA virus 3 (RsRV3/A105), Rhizoctonia solani partitivirus 3 (RsPV3/HG81), Rhizoctonia solani partitivirus 4 (RsPV4/HG81), and Rhizoctonia solani partitivirus 5 (RsPV5/C24), were also determined [35,36,38]. Furthermore, the complete genomes of three betapartitiviruses isolated from *R. solani* YNBB-111, Rhizoctonia solani partitivirus 6 to 8 (RsPV6-8/YNBB-111), were also characterized [37]. Moreover, the complete coding sequences of Rhizoctonia solani dsRNA virus 2 (RsDSRV2/A; Table 2) and Rhizoctonia solani partitivirus 6 to 8 (RsPV6/BR5, RsPV7/BR6 and RsPV8/BR16; Table S1) (RsPV6-8; Table S1), isolated from *R. solani* AG2-2 LP, have been determined using NGS. RsDSRV2/A, RsPV7/BR6 and RsPV8/BR16 RdRps belong to the genus *Alphapartitivirus*, while RsPV6/BR5 belongs to the genus *Betapartitivirus* [6]. Finally, a partial sequence of the Rhizoctonia solani partitivirus 1 from *R. solani* OA-1 has been determined (Table 2). In total, fourteen members of the family *Partitiviridae* have been found to infect *R. solani*, together with Rhizoctonia solani bipartite-like virus 1 (RsBPV1; Table 2), a member of the proposed family Bipartitiviridae [6].

#### 2.1.3. Unclassified dsRNA Viruses

To our knowledge, few studies characterized unclassified dsRNA viruses infecting *R. solani*. Rhizoctonia solani dsRNA virus 1 (RsRV1) (Figure 2; Table 2), found in the Chinese *R. solani* AG-1 IA B275 isolate from rice in 2007, was fully sequenced and analyzed. RsRV1 consists of two segments named RsRV1-dsRNA1 and RsRV1-dsRNA2, 2379 and 1811 bp in length, respectively, each containing a single open reading frame (ORF). RsRV1-dsRNA1 encodes an RNA-dependent RNA polymerase (RdRp; Pfam00680, E-value 1e-04), whereas RsRV1-dsRNA2 encodes a protein of unknown function. Both proteins are closely related to the unclassified Fusarium graminearum dsRNA mycovirus-4 (FgV-4) [16].

Rhizoctonia solani RNA virus HN008 (RsRV-HN008) (Figure 2; Table 2) was fully sequenced and characterized. RsRV-HN008 has a genome 7596 bp in length, containing two non-overlapping ORFs. ORF1 has no significant similarity to any protein in the databases, whereas ORF2 encodes an RdRp (Pfam02123, E-value 4e-14) with low similarity to that of Rosellinia necatrix megabirnavirus 1-W779 (RnMV1/W779; accession number LC333756, identity 29.06%, E-value 9e-71) [17].

M1 and M2 dsRNAs were found in *R. solani* Rhs 1A together with the genetically distinct dsRNAs L1 (25 kbp), L2 (23 kbp) and S1 (1.2 kbp), and represent the first well-described dsRNA elements in *R. solani* (Table 2) [40,62]. M1 is homologous to the recently described Rhizoctonia solani putative virus 1 (RsV1/BR4, Table 2; E-value 45.64%); it contains two putative ORFs on the positive strand, while four more have been reported on the negative strand [62]. M2 contains one main ORF which encodes an RdRp (Pfam05919; 4e-170) closely related to that of mitoviruses, such as the Rhizoctonia solani mitovirus 22 (RsMV22, Table S1; E-value 79.57%) and similar to the pentafunctional AROM polypeptide of the shikimate pathway, which synthesizes the five central steps of the shikimate pathway in filamentous fungi and yeast [40].

### 2.2. Single-Stranded RNA Viruses

In addition to dsRNA viruses, ssRNA viruses are also prevalent in *R. solani* [63]. Some viruses with the smallest and simplest genomes have ssRNA as their genetic material [64]. The ssRNA viruses may be classified as positive-sense (+) or negative-sense (−), based on the polarity of their RNA genome [65]. The (+)ssRNA viruses have a simple RNA replication and expression mechanism [66], while the (−)ssRNA viruses initiate replication by packaging their transcription and replication machinery into virions [67]. The majority of ssRNA mycoviruses reported have a linear monopartite (+)ssRNA genome [68]. According to the ICTV, (+)ssRNA mycoviruses are assigned in 8 families [69], including *Alphaflexiviridae* (5.4–9 kb in length), *Barnaviridae* (4 kb in length), *Botourmiaviridae* (2.9 kb in length), *Deltaflexiviridae* (6–8 kb in length), *Endornaviridae* (14–17.6 kb in length), *Gammaflexiviridae* (6.8 kb in length), *Hypoviridae* (9–13 kb in length) and *Narnaviridae* (1.7–2.9 kb in length). Only one (−)ssRNA mycovirus is officially recognized by the ICTV, Sclerotinia sclerotiorum negative-stranded RNA virus 1, which is closely related to nyaviruses and bornaviruses and was recently assigned to the family *Mymonaviridae* [70].

#### 2.2.1. (+)ssRNA Viruses: *Barnaviridae*

The *Barnaviridae* family currently accommodates genus *Barnavirus* and one species, *Mushroom bacilliform virus* [71] The exemplar of the species, mushroom bacilliform virus (MBV; accession number NC_001633) has a monopartite (+)ssRNA genome 4.0 kbp in length. The genome has four ORFs, encoding a protein of unknown function (P1), a polyprotein that includes protease and VPg domains (P2), RdRp (P3), and CP (P4), respectively. Few viruses related to genus *Barnavirus* have been discovered so far, including Colobanthus quitensis associated barnavirus 1 (CqBV1; accession number MG686618) and Rhizoctonia solani barnavirus 1 (RsBV1) [19]. RsBarV1 is 3915 bp in length and contains three ORFs, encoding a polyprotein with protease (Pfam02122, E-value 9e-06) and VPg domains, an RdRp (Pfam02123; E-value 5e-30) similar to that of MBV (identity 47%, E-value 1e-124), and a CP. The ORF encoding the protein of unknown function is missing, suggesting that the 5′ terminal sequence of RsBV1 is incomplete.

#### 2.2.2. (+)ssRNA Viruses: *Benyviridae*

The family *Benyviridae* accommodates (+)ssRNA plant viruses with rod-shaped virions, whose genome is capped and polyadenylated, comprises four to five segments and ranges from 1.3 to 6.7 kb in length [46]. *Benyviridae* accommodates four species within the genus *Benyvirus* and its members are associated with cell-to-cell movement [46,72,73]. Two distinct viruses, both named Rhizoctonia solani Beny-like virus 1 (RsBenV1; Table 2) were found in *R. solani* 42304-9a [19] and *R. solani* AG-2.2 LP BR2 [6], respectively, and were partially characterized. In each case, only one segment of the genome was identified, encoding a putative RdRp related to benyviruses and benylike-viruses; more specifically RsBenV1/42304-9a is closely related to beet soil-borne mosaic virus (BSBMV; accession number AF280539, identity 39.13%, E-value 1e-09), an official member of the *Benyviridae* family, while RsBenV1/BR2 is closely related to Sclerotium rolfsii beny-like virus 1 (SrBLV1; accession number MH766487, identity 40.74%, E-value 0.0).

#### 2.2.3. (+)ssRNA Viruses: *Botourmiaviridae* and Basidionarnaviridae

*Botourmiaviridae* is a family of plant and fungal viruses with (+)ssRNA genomes. The family *Botourmiaviridae* currently accommodates ten species and four genera: *Botoulivirus* (1 segment, 2.9 kbp in length), *Magoulivirus* (1 segment, 2.3 kbp in length), *Ourmiavirus* (3 segments, approximately 0.9 kbp, 1.0 kbp and 2.8 kbp in length, respectively), and *Scleroulivirus* (1 segment, 3 kbp in length). NGS has led to the identification of new viruses infecting *R. solani* which are related to *Botourmiaviridae*, Rhizoctonia solani ourmia-like virus (RsOLV) 1-5 [6,19]. Only 59%–87% of the RsOLV1 genome was sequenced and the original analysis revealed similarity to the RdRps of members of the genus *Ourmiavirus* such as Cassava virus C (CVC; accession number NC_013111, identity 33.73%, E-value 2e-07), Epirus cherry virus (EcV; accession number NC_011065, identity 33.61%, E-value 2e-09) and Ourmia melon virus (OmV; accession number NC_011068, identity 32.61%, E-value 9e-09) [19].

**Figure 3:**
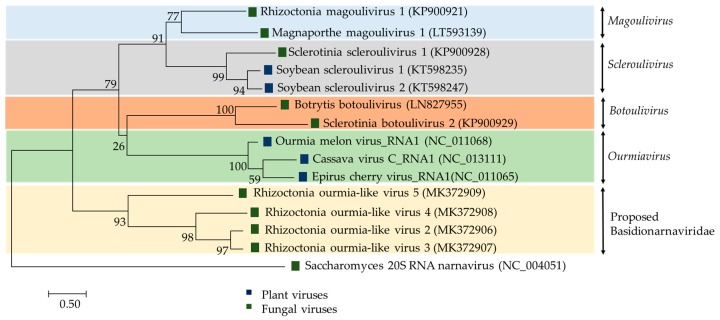
Phylogenetic analysis of viruses related to the family *Botourmiaviridae*. The phylogenetic tree was built using the maximum likelihood method; substitution model LG+G+I. 1000 bootstrap replications were applied.

Plant viruses of the genus *Ourmiavirus* are tripartite with each segment encoding a single protein: RdRp, CP and movement protein (MP), respectively, and are believed to have evolved by reassorting genomic segments of viruses infecting fungi and plants [74]. In contrast, the RsOLV1 genome does not appear to encode the CP or the MP [19]. Currently, RsOLV1 is the exemplar of the officially recognized species *Rhizoctonia magoulivirus 1*, genus *Magoulivirus*, family *Botourmiaviridae*. In contrast, evolutionary phylogenetic tree clustered RsOLV 2 to 5 together Agaricus bisporus virus 15 (AbV15/003; accession number AQM49945) into a potential novel genus within the family *Botourmiaviridae* or even a novel closely related family (Figure 3) provisionally named Basidionarnaviridae since it currently accommodates only viruses infecting basidiomycetes [6]. The RsOLV2/Rs, RsOLV2, and RsOLV3 RdRp sequences are over 70% identical, therefore they are likely different isolates of the same species [6].

#### 2.2.4. (+)ssRNA Viruses: *Bromoviridae*

*Bromoviridae* is a family of viruses with worldwide distribution that naturally infects plants. There are currently six genera in the family, including *Alfamovirus*, *Anulavirus*, *Bromovirus*, *Cucumovirus*, *Ilarvirus*, and *Oleavirus*. *Bromoviridae* possess a tripartite linear (+)ssRNA genome [75], approximately 8 kb in length [76]. RNA1 and RNA2 encode RdRp 1a and 2a, respectively, both involved in genome replication and transcription of ssRNA4 from the minus-strand copy of RNA3. RNA3 produces a MP and a CP. Members of the genera *Cucumovirus* and *Ilarvirus* have an additional overlapping ORF [75]. Members of the family *Bromoviridae* form virions, either spherical or quasi-spherical for the members of the genera *Cucumovirus*, *Ilarvirus*, *Anulavirus*, and *Bromovirus*, or bacilliform for the members of the genera *Ilarvirus*, *Alfamovirus*, and *Oleavirus* [76].

**Figure 4:**
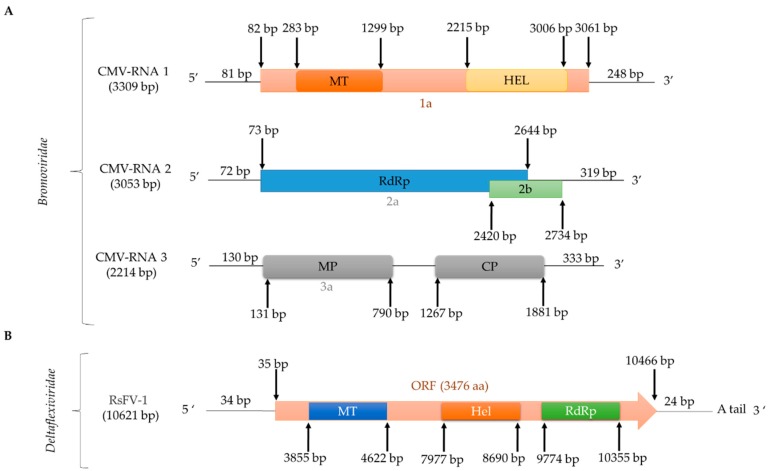
Schematic representation of the genomic organization of single-stranded (ss)RNA viruses infecting *R. solani*: (**A**) bromovirus CMV; CMV-RNA 1 encodes 1a (replicase component; Pfam12467, E-value 1e-73), CMV-RNA 2 encodes 2a (replicase component; Pfam00978, E-value 0.0) and 2b (RNA-silencing suppressor), and CMV-RNA 3 encodes 3a (MP; Pfam00803, E-value 1e-88) and coat protein (CP); (**B**) deltaflexivirus RsFV-1/DC717.

Natural cross-kingdom virus transmission between plants and fungi has been speculated for some time and recently transmission of CMV to *R. solani* was reported [7]. CMV-infected *R. solani* AG-3 (Figure 4) was isolated from potato plants (*Solanum tuberosum* L.) in Inner Mongolia, China. CMV transmission can occur in both directions from plant to *R. solani* and *R. solani* to plant, while CMV can be transmitted horizontally via hyphal fusion but not vertically via basidiospores [7]. CMV is a member of the genus *Cucumovirus*, family *Bromoviridae* [7] and has three genomic segments 3309 nt, 3053 nt, and 2214 nt in length, respectively, encapsidated in isometric particles [7].

#### 2.2.5. (+)ssRNA Viruses: *Deltaflexiviridae* and *Tymoviridae*, *Tymovirales*

The general term flexiviruses refers to members of the order *Tymovirales*, families *Alphaflexiviridae*, *Betaflexiviridae*, *Deltaflexiviridae*, and *Gammaflexiviridae*. Flexiviruses have a monopartite (+)ssRNA polyadenylated genome 6.5–9.5 kb in length and filamentous virions, which encode a replication-associated polyprotein 150–250 kDa in size [68] and are known to infect both plants and fungi [77,78]. The first mycovirus reported in the order *Tymovirales* was Botrytis virus F (BotV-F), which belongs to the family *Gammaflexiviridae*, genus *Mycoflexivirus* [79]. Within the family *Deltaflexiviridae*, three species belonging to the genus *Deltaflexivirus* have been reported: Sclerotinia deltaflexivirus 1 (SsDFV1) [80], soybean-associated deltaflexivirus 1 (SlaMFV1) [81], and Fusarium deltaflexivirus 1 (FgDFV1) [68]. Only one flexivirus infecting *R. solani* has been fully sequenced in, tentatively named Rhizoctonia solani flexivirus 1 (RsFV1; Figure 4; Table 2) [15]. RsFV-1 was isolated from *R. solani* AG2-2IV/DC17 and its (+)ssRNA genome consists of 10,621 nt excluding the poly (A) tail. RsFV-1 encodes a single protein similar to that of other members of the order *Tymovirales*, most notably the deltaflexiviruses SsDFV1 (accession number NC_038977, identity 35.5%, E-value 2e-104), SlaMFV1 (accession number NC_038979, identity 34.32%, E-value 9e-110) and FgDFV1 (accession number NC_030654, identity 38.8 %, E-value 2e-111) [15]. Additionally, the RsFV-1 protein has three conserved domains, including a viral methyltransferase (Pfam01660, E-value 3.65e-29), a viral helicase (Pfam01443, E-value 2.04e-08), and an RdRp (Pfam00978, E-value 2.46e-08). Two more flexiviruses, Rhizoctonia solani flexivirus 2 (RsFV2; Table S1) and Rhizoctonia solani flexi-like virus 1 (RsFLV1; Table 2) were detected in *R. solani* AG2-2IV/DC17 and *R. solani* AG-2.2 LP BR9, respectively, and have been partially sequenced.

*Tymoviridae* is a family of (+)ssRNA viruses in the order *Tymovirales* which range from 6.0 to 7.5 kb in length [82]. The family *Tymoviridae* currently accommodates three genera *Maculavirus*, *Marafivirus*, and *Tymovirus* and 41 officially recognized species [69]. However, more viruses related to *Tymoviridae* have been reported but not classified thus far [76], including Rhizoctonia solani positive-stranded RNA virus 1 (RsPSV1; Figure 2) [19], the only known tymovirus infecting *R. solani*. The partial genome sequence of RsPSV1 contains a large ORF and several small ORFs, similar to the bee Macula-like virus (MlV; accession number NC_027631, identity 30%, E-value 4e-56) in the family *Tymoviridae*.

#### 2.2.6. (+)ssRNA Viruses: *Endornaviridae*

*Endornaviridae* is a family of viruses with non-encapsidated RNA genomes that range in size from 9.7–17.6 kb and contains a single ORF encoding a polyprotein [83,84]; the polyprotein has an RNA helicase domain at the N-terminus and conserved RdRp motifs at the C-terminus [85]. Endornaviruses naturally infect fungi, plants, and oomycetes, which are persistent and do not cause obvious symptoms in their host [85,86,87]. In fungal hosts, endornaviruses are transmitted vertically via sporulation and horizontally via anastomosis [88], whereas in plant hosts they rely on vertical transmission via pollen and ova, since they lack a MP and cannot move from cell to cell [89,90,91]. Endornaviruses are not encapsidated and do not form true viral particles [85]. The family accommodates two genera, *Alphaendornavirus* and *Betaendornavirus*.

An endornavirus, tentatively named Rhizoctonia solani endornavirus (Table 2) and isolated from *R. solani* AG-3PT strain RS002 (RsEV-RS002), was partially sequenced. The RsEV-RS002 partial genome (14964 nt) includes a partial 5′ untranslated region (5′-UTR) but not the 3′-UTR. The RsEV-RS002 genome shows low similarity to the genomic sequence of bell pepper alphaendornavirus (BPEV-YW; accession number NC_015781, identity 29.8%, E-value 1e-71). A conserved domain search in Pfam [92] showed that the RsEV-RS002 protein has three conserved domain motifs including a viral methyltransferase (MT; Pfam01660, E-value 5e-05), a viral helicase (Hel; Pfam01443, E-value 7e-11), both located at the N-terminus, and an RdRp (Pfam00978, E-value 3e-16) located at the C-terminus. The putative RdRp domain is located at the C-terminus whereas the putative viral helicase and MT are both located at the N-terminus [14]. Rhizoctonia solani endornavirus 1 (RsEV1/GD-2; Table S1), Rhizoctonia solani endornavirus 2 (RsEV2/Illinois1; Table 2), Rhizoctonia solani endornavirus 3 (RsEV3/DC17; Table S1), and Rhizoctonia solani endornavirus 4 to 7 (RsEV4-7; Table S1) have also been reported [6]. RsEV4, 5, 6, and 7 each contain a single putative ORF of 6719, 5300, 5077 and 4757 aa, respectively. RsEV4, RsEV6 and RsEV7 encode an RdRp (Pfam00978; E-value 9e-23, 9e-22, and 1e-25, respectively) and a Hel (Pfam01443; E-value 7e-12, 3e-06, and 1e-25, respectively) domain. RsEV5 encodes an MT (Pfam01660; E-value 4e-05) domain in addition to the RdRp (Pfam00978; E-value 5e-17); however, no Hel domain was detected. Additionally, a complete genome sequence of an endornavirus from *R. cerealis*, another species of the genus *Rhizoctonia*, has been described [93] and is the exemplar of an officially recognized species *Rhizoctonia cerealis alphaendornavirus 1*. RsEV-RS002, RsEV1/GD-2 and RsEV5 belong to genus *Alphaendornavirus* as well, while a new genus Gammaendornavirus within the family *Endornaviridae* was recently proposed to accommodate RsEV2/Illinois1, RsEV3/DC17, RsEV4, RsEV6, and RsEV7 [6].

#### 2.2.7. (+)ssRNA Viruses: *Hypoviridae* and Fusariviridae

The family *Hypoviridae* accommodates a single genus, *Hypovirus*, and four recognized species, *Cryphonectria hypovirus 1* to *4*, with capsidless monosegmented (+)ssRNA genomes ranging from 12.7 to 9.2 Kbp in length [94,95,96,97,98]. Each genome has either one or two ORFs, encoding at least putative RdRp and Hel domains [98] and occasionally additional domains including glucosyltransferase (UGT), papain like protease (PRO) and permuted papain-fold peptidase of dsRNA viruses and eukaryotes (PPPDE) [99,100]. The primary interest in hypoviruses stemmed from their ability to mitigate the fungal host virulence (hypovirulence), of the chestnut blight fungus *Cryphonectria parasitica*. Hypoviruses can be transmitted horizontally to virulent isolates via hyphal anastomosis [99].

To our knowledge, a complete hypovirus genome from *R. solani* has not been reported so far, but the complete ORF of Rhizoctonia solani hypovirus 1 (RsHV1; Table 2) and partial ORFs for Rhizoctonia solani hypovirus 2 and 3 (RsHV2 and 3, respectively; Table S1) were recently described using NGS [6]. The RsHV1 segment is 18 kbp in length, representing one of the longest hypovirus genomes known so far, and encodes a large putative protein of 5344 aa where only a helicase conserved domain (cd00046, E-value 6.99e-06) was detected; neither an RdRp domain nor the GDD motif, hallmark of most viral RdRps, was found in the protein sequence. Nevertheless, BLAST analysis revealed that the RsHV1 protein was homologous to other hypoviruses such as Sclerotinia sclerotiorum hypovirus 2 (SsHV2; accession number QBA69886, identity 26.64%, E-value 4e-81) [101]. The RsHV2 and 3 sequences are 9 and 5 kbp in length, respectively, with two ORFs each. The two RsHV2 ORFs encode proteins homologous to those of hypoviruses but lacking any conserved motifs, while one of the RsHV3 ORFs has a helicase conserved domain (cd00046, E-value 3.62e-10). A new genus Megahypovirus within the family *Hypoviridae* was proposed to accommodate RsHV1 and SsHV2, whose genomes are large, together with Agaricus bisporus virus 2 (AbV2/003; accession number KY357506), RsHV2 and RsHV3 [6].

Furthermore, three fusariviruses Rhizoctonia solani fusarivirus 1, 2 and 3 (RsFV1, 2 and 3, respectively) were described [6] related to the members of the proposed family Fusariviridae [102]. The RsFV1 genomic segment is 11 kbp in length containing four putative ORFs: the largest ORF3 encodes a protein with an RdRp (Pfam00680; E-value 7e-20) and a Hel (Pfam00270; E-value 1.2e-06) domain; ORF1 encodes a viral helicase (Pfam04851; E-value 3.7e-09); the smallest ORF2 and ORF4 encode proteins of unknown function. RsFV2 has similar genomic organization, while the RsFV3 partial genomic sequence encodes for an RdRp (Pfam00680; E-value 1.2e-06) and a Hel (cd00046; E-value 2e-06) domain [6]. It has been proposed to subdivide the currently described fusariviruses into at least two further genera, based on the sequence length and genome organization, and in this case RsFV1 and RsFV2 would cluster together while RsFV3 would belong to a different genus [6].

#### 2.2.8. (+)ssRNA Viruses: *Narnaviridae*

Members of the family *Narnaviridae* are the simplest viruses with a linear (+)ssRNA genome 1.7–3.6 kb in length, a single ORF, which encodes an RdRp [103,104], and no capsid. The family *Narnaviridae* accommodates two genera, *Mitovirus* and *Narnavirus* [103]. All known members of genus *Mitovirus* infect filamentous fungi and plants [104,105], whereas members of the genus *Narnavirus* have been also found in the yeast *Saccharomyces cerevisiae* and in the oomycete *Phytophthora infestans* [43]. Mitoviruses have genomes 2.3–3.1 kb in length [106] and do not form true virions but are associated with lipid membrane-bound vesicles [107]. Mitoviruses replicate in the mitochondria of the host cell, in contrast to narnaviruses that are known to replicate in the cytosol. Since the discovery of the first mitovirus in *C. parasitica*, many mitoviruses have been detected in phytopathogenic fungi [106], most of them from ascomycetes, a few from basidiomycetes and one from arbuscular mycorrhiza [108]. Some mitoviruses have been reported to confer hypovirulence to their host, such as Sclerotinia sclerotiorum mitovirus 4 (SsMV4) isolated from *S. sclerotiorum* strain AH16 [109].

To our knowledge, there are no complete mitovirus genomes described from *R. solani*, however, forty partial genome sequences of mitoviruses infecting *R. solani* have been reported [6,9,21]. A novel mitovirus infecting *R. solani* AG-3PT strain RS002 [18] was characterized and tentatively named Rhizoctonia solani mitovirus 1 (RMV1-RS002; Table 2). The partial genome sequence of RMV1-RS002 is 2797 nt and shows similarity to the tuber excavatum mitovirus (TeMV; accession number JN222389, identity 25.6%, E-value 4e-106) [18]. The protein encoded by RMV1-RS002 is similar to Glomus sp. RF1 small virus (GRF1V-S; accession number NC_040656, identity 49.55%, E-value 7e-94) [18]. The partial 5′-UTR of RMV1-RS002 was shown to form at least three stem-loop structures [18], as is typical for viral UTRs in general. In addition to of RMV1-RS002, several partial mitovirus genomes sequences have been reported (Table S1) [6,9,19]. A new family Mitoviridae, closely related to but distinct from the family *Narnaviridae*, has been proposed to accommodate current members of the genus *Mitovirus* and other mitoviruses. This new family would be subdivided into a number of genera, including plant and fungal mitoviruses [6,105,110]. Moreover, a new order would be established to include the *Narnaviridae* and the proposed Mitoviridae families [95].

#### 2.2.9. (+)ssRNA Viruses: Mycoalphaviridae

The *Togaviridae* family accommodates genus *Alphavirus* and 31 species, including several important human pathogens such as Eastern equine encephalitis virus (EEEV) [111], Western equine encephalitis virus (WEEV) [112], Venezuelan equine encephalitis virus (VEEV) [113], Sindbis virus (SINV) [114], Ross River virus (RRV) [115], Semliki Forest virus (SFV) [116], and Chikungunya virus [117]. Alphaviruses are arboviruses that are transmitted alternatively between insect vectors and vertebrate hosts [118,119]. Members of the *Togaviridae* family are small enveloped (+)ssRNA viruses ranging from 10 to 2 Kbp in size [119], with a methylguanosine cap and a poly(A) stretch at the 5′ end and 3′ end, respectively, and a genome encoding both non-structural and structural proteins [118]. The virion consists of a nucleocapsid core, a lipid bilayer and surface glycoproteins [120].

To our knowledge, there is no member of the genus *Alphavirus* infecting *R. solani*. However, partial genomic sequences related to *Togaviridae* family were recently reported in *R. solani* AG-2.2 LP, including Rhizoctonia solani alphavirus-like 1, 2, and 3 (RsALV1/BR15, Table 2; RsALV2/BR14 and RsALV3/BR8, Table S1) [6]. The RsAVL2 partial ORF encodes RdRp (Pfam00978, E-value 2.9e-21), whereas both RdRp (Pfam00978, E-values 3.8e-18 and 2.1e-19, respectively) and viral helicase (Pfam01443, E-values 8.5e-05 and 6.6e-11, respectively) domains can be detected in RsAVL1 and RsAVL3 [6]. A new family Mycoalphaviridae was proposed to accommodate RsALV1, RsALV 2, and RsALV3, together with Rhizoctonia solani RNA virus 1, 2, and 3 (RsRV1-3/DC17; Table S1) detected in *R. solani* AG 2-2IV DC17 and Sclerotinia sclerotiorum RNA virus L (SsRVL; accession number EU779934) [6].

#### 2.2.10. (−)ssRNA Viruses: Betamycoserpentoviridae, *Serpentovirales*

*Aspiviridae*, formerly known as *Ophioviridae*, is a family of flexible filamentous viruses known to infect plants [121] and belongs to the order *Serpentovirales*. The family *Aspiviridae* currently accommodates one genus *Ophiovirus* and 7 species. The members of the family *Aspirividae* contain a (−)ssRNA genome ranging from 11.3 to 12.5 kb in length separated into 3 to 4 segments [121]. Recently, unclassified partial virus sequences related to ophioviruses were reported infecting soil-borne *R. solani* strains and named Rhizoctonia solani negative-stranded RNA virus 1 to 3 (RsNSRV1-3; Table 2). Analysis of the sequences revealed a large ORF with significant similarity to the L proteins encoded by RNA1 of the lettuce ring necrosis ophiovirus and other members of the family *Aspiviridae*. A new family Betamycoserpentoviridae within the order *Serpentovirales* has been proposed to accommodate these viruses, together with Fusarium poae negative-stranded RNA virus 1 (FpNSV1) from the fungal plant pathogen *Fusarium poae* [19,34].

#### 2.2.11. (−)ssRNA Viruses: Mycophleboviridae, *Bunyavirales*

The order *Bunyavirales* accommodates twelve families: *Arenaviridae*, *Cruliviridae*, *Fimoviridae*, *Hantaviridae*, *Leishbuviridae*, *Mypoviridae*, *Nairoviridae*, *Peribunyaviridae*, *Phasmaviridae*, *Phenuiviridae*, *Tospoviridae*, and *Wupedeviridae*. Metatranscriptomics analyses of plant pathogenic fungi revealed the presence of several (−)ssRNA mycoviruses, related to bi- and tri-segmented (−)ssRNA viruses, such as peribunyaviruses and phenuiviruses [19,33,34]. For instance, Lentinula edodes negative-strand RNA virus 2 (LeNSRV2) infecting Lentinula edodes is a phenui-like virus and the first segmented (−)ssRNA virus found to infect fungi [28], while more viruses related to the order *Bunyavirales* were reported in fungi associated with the marine organism Holothuria polii [29] and the ascomycete Entoleuca sp. [27]. Recently, two viruses infecting *R. solani* and related to bunyaviruses were reported: Rhizoctonia solani bunya/phlebo-like virus 1 (RsBPLV1; Table 2) [6] and Rhizoctonia solani negative-stranded virus 4 (RsNSV4; Table S1). Analysis of the protein encoded by the RsBPLV1 segment revealed the presence of RdRp motifs (Pfam04196; E-value 1e-09). Subsequently, the new family Mycophleboviridae was proposed within the order *Bunyavirales* to accommodate RsBPLV1 and RsNSRV1 together with Ixodes scapularis associated virus 6 (IsV6; accession number MG256514).

## 3. Transmission of Viruses Infecting *Rhizoctonia solani*

Mycovirus transmission is a significant process that needs to be addressed in any mycovirus-mediated biological control approach to alleviate fungal diseases. Specifically, it is necessary for the mycovirus to acquire some functions before being considered as a potential biological control agent, including limitation of host range to prevent the spread to undesirable hosts and the ability to establish and spread within the targeted host population [122]. Two principal pathways of transmission are known: Horizontal transmission via hyphal anastomosis and heterokaryosis, and vertical transmission through sporulation [123]. The effectiveness and success of biological control may vary depending on the mycovirus mode of transmission. Horizontal transmission is generally linked to increased biocontrol efficiency, since it leads to widespread coverage of the biocontrol agent, whereas vertical transmission is associated with lower efficiency [124]. Mycoviruses are completely dependent on their host due to their inability to survive in the environment and vertical transmission may have evolved in cases of mutualism. Nevertheless, some cases of horizontal transmission in mutualistic symbiosis have been reported [125]. The replication cycle of mycoviruses, in general, lacks an extracellular phase and infectious virions; one notable exception is the novel circular ssDNA virus, Sclerotinia sclerotiorum hypovirulence associated DNA virus 1 (SsHADV-1), which can be transmitted extracellularly and use a mycophagous insect (*Lycoriella ingénue*) as a vector for transmission [43,106]. This suggests the potential existence of other undiscovered mycoviruses that might be transmitted extracellularly.

In *R. solani*, few studies have been reported on mycovirus transmission. Successful transfection protocols were previously established for some mycoviruses, including members of the families *Partitiviridae*, *Megabirnaviridae*, *Reoviridae*, and *Totiviridae*. This approach is generally based on the use of polyethylene glycol (PEG) 4000 that promotes protoplast fusion and subsequent regeneration of the virus-transfected protoplasts and contributes substantially to the understanding of virus-host interaction and mycovirus-mediated biological control [17]. For instance, the alphapartitivirus RsPV2/GD-11 was successfully introduced into protoplasts of the virus-free *R. solani* strain GD-118 creating the derivative virus-transfected strain GD-118T [17]. Despite the complexity of fungal cell walls which are considered to be a substantial barrier to their spread, mycoviruses are generally capable of transmission from one fungal isolate to another in nature [25]. Purified RsPV2/GD-11 particles were successfully transmitted horizontally or vertically, although in some cases transmission via hyphal fusion failed between different genotypes within the same *R. solani* anastomosis group [22]. In addition, members of the family *Endornaviridae*, which do not produce virus particles, are transmissible at high rates horizontally as well as vertically [14]. For instance, a betaendornavirus identified in *R. solani* Ra1 has the ability to be transmitted vertically via basidiospores [7], while the alphaendornavirus RsEV1/GD-2 could be transmitted horizontally via hyphal anastomosis [39]. Furthermore, the M2 dsRNA and the betapartitiviruses RsPV6/YNBB-111, RsPV7/YNBB-111 and RsPV8/YNBB-111 could be transmitted horizontally via hyphal anastomiosis [37,40]. Moreover, CMV infecting *R. solani* was transmitted horizontally through hyphal fusion, but not vertically via basidiospores [7].

## 4. Effects of Virus Infection on *Rhizoctonia solani*

Mycovirus infections are often cryptic (symptomless) but investigations focus on potential hypovirulence, a phenomenon that may be exploited in the context of sustainable biological control of fungal diseases. The prime example is Cryphonectria hypovirus 1 (CHV1), used to successfully control the plant pathogen *Cryphonectria parasitica*, the causal agent of chestnut blight, in Europe [122]. This discovery revolutionized the world of fungal biological control and led to the term hypovirulence [126]. Additionally, Rosellinia necatrix megabirnavirus 1 (RnMBV1) was isolated from *Rosellinia necatrix* the causative agent of a worldwide devastating disease. RnMBV1 belongs to the family *Megabirnaviridae*, has a bi-segmented genome and is a potential virocontrol agent since it confers hypovirulence by significantly reducing the virulence and mycelial growth of its host [53,69]. The main effects include a decrease in the host growth rate, attenuation of host virulence, lack of sporulation and reduction of basidiospore germination [127,128]. In addition, other mycoviruses may have more deleterious effects, including the ‘La France’ disease of *Agaricus biporus* caused by the ‘La France’ isometric virus (LIV) and the mushroom diseases caused by oyster mushroom isometric virus (OMIV) and oyster mushroom spherical virus (OMSV) [21]. To investigate the effect of mycoviruses on their hosts it is important to construct a virus-free isogenic line, either by transmitting the mycovirus into a virus-free strain or by curing the virus-infected one [25]. For instance, protoplast transfection of RsPV2/GD-11 into the *R. solani* virus-free strain GD-118 resulted in darker mycelial pigmentation on potato dextrose agar (PDA) plates, and a reduction of mycelial growth rate, sclerotia size and numbers [17]. Furthermore, RsPV2/GD-11 diminished lesion sizes on rice leaves, indicating hypovirulence [17]. Similarly, horizontal transmission of RsEV5/GD-2 resulted in host hypovirulence [39]. In contrast to RsPV2/GD-11 and RsEV5/GD-2, infection of *R. solani* with CMV does not affect the growth rate and morphology of the fungus on PDA under laboratory conditions [7]. Additionally, M1 dsRNA is associated with enhanced virulence in the parental *R. solani* Rhs 1A, while sectors of the parental strain harboring the M2 dsRNA and the derivative strains showed reduced pigmentation and growth rate [41]. The RNA titers of M1 and M2 dsRNAs appear to be inversely correlated [40]; the former can be found mainly in mitochondria [62] while the latter in the cytosol [40]. All these studies clearly illustrate the phenotypic variation of mycovirus infection.

## 5. Conclusions and Future Prospects

Viruses infecting *R. solani* are less well studied as compared to those in other fungal genera such as *C. parasitica*. However, a range of RNA viruses infecting *R. solani* was described including members of the families *Barnaviridae, Benyviridae*, *Botourmiaviridae*, *Bromoviridae*, *Deltaflexiviridae*, *Endornaviridae*, *Hypoviridae*, *Megabirnaviridae*, *Narnaviridae*, *Partitiviridae*, *Togaviridae*, and *Tymoviridae*, together with unclassified mycoviruses related to the orders *Serpentovirales* and *Bunyavirales*. These families include dsRNA viruses, (+)ssRNA viruses and (−)ssRNA viruses and the majority of the viruses infecting *R. solani* have dsRNA or (+)ssRNA genomes.

In addition to the discovery of novel viruses, future research on mycoviruses needs to focus on the molecular mechanisms of mycovirus–host interactions and provide a better understanding of mycovirus transmission mechanisms. Efficient mycovirus detection relies on NGS technology. NGS allows the determination of mycoviruses previously unreported and contributes considerably to the clarification of unknown molecular mechanisms of host-virus interactions since it can be used to examine in detail changes in the *R. solani* transcriptome following mycovirus infection. Viruses infecting *R. solani* are transmissible horizontally via anastomosis hyphal fusion or vertically via sporulation [129], while successful transmission depends on the particular mycovirus under study. For example, endornaviruses use both routes of transmission, horizontal and vertical [14], whereas CMV was efficiently transmitted horizontally in *R. solani* CMV-free strains via hyphal fusion but failed to transmit through basidiospores [7]. No specific vectors facilitating mycovirus transmission have been reported although it is believed that yet undetermined insect vectors may play a key role and these should be identified in the future. Mycovirus-related research focuses especially on the identification of potential biological agents to combat plant pathogenic fungi. In the case of *R. solani*, some viruses such as CMV have no discernible effects on their host, while others such as RsPV2/GD-11 were shown to cause hypovirulence and therefore are promising biocontrol agents and should be studied extensively in the future.

## Figures and Tables

**Table 1:** *R. solani* anastomosis groups (AGs) and subgroups with their reported hosts or habitats.

Anastomosis Groups (AGs)	Anastomosis Subgroups	Host or Habitat
AG-1	1-IA, 1-IB, 1-IC, 1-ID, 1-IE, 1-IF	Rice, maize, soybean *Brassica* crops, Sudan grass
AG-2	2-1, 2-1 II, 2-2 IIIB, 2-2 LP, 2-3	Tobacco, *Brassica* crops, six-rowed barley, wheat, rice, grass
AG-3	3 IV	Tobacco, potato, *Brassica* crops
AG-4	4-HGI, 4-HGII, 4-HGIII, AGIIIA	Potato, *Brassica* crops, cauliflower
AG-5		*Brassica* crops, soil
AG-6		*Brassica* crops, soil
AG-7		*Brassica* crops, radish field soil
AG-8		*Brassica* crops
AG-9		*Brassica* crops
AG-10		*Brassica* crops
AG-11		*Brassica* crops
AG-12		*Brassica* crops
AG-13		*Brassica* crops
AGBI		Soil

**Table 2:** Representative viruses known to infect *Rhizoctonia solani*.

Name (abbr.) Rhizoctonia solani	Classification (Family)	Host Strain	Segment	Genome Size (bp/nt)	5′ UTR (bp/nt)	3′ UTR (bp/nt)	ORF Length (bp/nt)	Protein Length (aa)	Molecular Mass (kDa)	Accession Number
barnavirus 1 (RsBarV1)	*Barnaviridae*	DV-8	(+)ssRNA	3914 partial	≥69	≥176	2033	677	75.9	KP900904
							1424	474	53.1	
							557	185	20.7	
beny-like virus 1 42304-9a (RsBenV1/42304-9a)	*Benyviridae*	42304-9a	(+)ssRNA	1306 partial	≥1	≥1	≥1306	≥435	≥48.6	KP900902
beny-like virus 1 BR2 (RsBenV1/BR2)	*Benyviridae*	AG-2.2 LP BR2	(+)ssRNA	11666 partial	≥622	≥289	10755	3584	403.9	MK507778
ourmia-like virus 1 (RsOLV1)	*Botourmiaviridae*	RsAG2	(+)ssRNA	2792 partial	≥1	≥689	≥2103	≥700	≥79.0	KP900921
Cucumber mosaic virus (CMV)	*Bromoviridae*	AG-3	(+)ssRNA	3309	81	248	2959	992	111.3	MG025947
			(+)ssRNA	3053	72	316	2573	856	96.5	MG025948
							332	62	7.27	
			(+)ssRNA	2214	97	321	839	278	30.3	MG025949
							656	218	23.8	
flexivirus 1 (RsFV1)	*Deltaflexiviridae*	AG2-2 IV DC17	(+)ssRNA	10644	34	176	10,433	3476	381.0	KX349055
flexi-like virus 1 (RsFLV1)	*Deltaflexiviridae*	AG-2.2 LP BR9	(+)ssRNA	2982 partial	≥15	≥178	2888	962	110.8	MK507787
endornavirus RS002 (RsEV-RS002)	*Endornaviridae*	AG-3PT RS002	(+)ssRNA	14694 partial	≥13	≥1	≥14,680	≥4893	≥555.6	KC792590
endornavirus 2 Illinois1 (RsEV2/Illinois1)	*Endornaviridae*	Illinois1	(+)ssRNA	15850 partial	≥35	≥26	15,783	5262	597.0	KT823701
hypovirus 1 (RsHV1)	*Hypoviridae*	AG-2.2 LP BR20	(+)ssRNA	18371 partial	≥752	≥1584	16,033	5344	363.0	MK558259
megabirnavirus 1 (RsMBV1)	*Megabirnaviridae*	AG2-2 IV DC17	dsRNA	975 partial	≥1	≥1	≥975	≥325	≥36.2	KX349071
mitovirus 1 RS002 (RMV1-RS002)	*Narnaviridae*	AG-3PT RS002	(+)ssRNA	2797 partial	≥192	≥126	2475	825	92.7	KC792591
virus 717 (RsV-717)	*Partitiviridae*	AG-2 Rhs 717	dsRNA1	2363	85	88	2189	730	86.0	AF133290
			dsRNA2	2206	78	76	2051	683	76.0	AF133291
partitivirus 1 OA-1 (RsPV1/OA-1)	*Partitiviridae*	OA-1	dsRNA1	1810 partial	≥1	≥1	≥1810	≥603	≥67.3	KU299048
partitivirus 2 GD-11 (RsPV2/GD-11)	*Partitiviridae*	AG-1 IA GD-11	dsRNA1	2020	88	60	1871	623	72.6	KF372436
			dsRNA2	1790	107	213	1469	489	53.3	KF372437
dsRNA virus 2 A (RsDSRV2/A)	*Partitiviridae*	AG-2.2 LP A	dsRNA1	1942 partial	≥58	≥11	1869	622	76.6	MK400668
			dsRNA2	1727 partial	≥79	≥181	1467	488	53.3	MK400669
positive-stranded RNA virus 1 (RsPSV1)	*Tymoviridae*	Illinois1	(+)ssRNA	3492 partial	≥1	≥248	≥2265	≥754	≥85.0	KT823702
							542	180	20.2	
							596	198	22.2	
bipartite-like virus 1 (RsBLV1)	Bipartitiviridae	AG-2.2 LP BR1	dsRNA	1827 partial	≥39	≥1	1787	595	68.5	MK492913
				1888 partial	≥126	≥151	972	323	37.0	MK492914
							552	183	21.1	
negative-stranded RNA virus 1 (RsNSRV1)	Betamycoserpentoviridae	DK13-1	(−)ssRNA	5593 partial	≥148	≥1	≥7237	≥2411	≥271.0	KP900919
negative-stranded RNA virus 2 (RsNSRV2)	Betamycoserpentoviridae	248-36	(−)ssRNA	7335 partial	≥136	≥192	7145	2381	267.6	KP900920
negative-stranded RNA virus 3 (RsNSRV2)	Betamycoserpentoviridae	DK13-3	(−)ssRNA	7335 partial	≥127	≥65	7142	2380	267.5	KP900903
fusarivirus 1 BR18 (RsFV1/BR18)	Fusariviridae	AG-2.2 LP BR18	(+)ssRNA	10776 partial	≥161	≥235	2194	731	49.7	MK558257
							1577	525	35.7	
							4682	1560	106.0	
alphavirus-like 1 BR15 (RsALV1/BR15)	Mycoalphaviridae	AG-2.2 LP BR15	(+)ssRNA	2414 partial	≥61	≥1	≥2352	≥784	≥90.3	MK507793
bunya/phlebo-like virus 1 (RsBPLV1)	Mycophleboviridae	AG-2.2 LP BR3	(−)ssRNA	7804 partial	≥150	≥112	7542	2513	295.6	MK507779
RNA virus HN008 (RsRV-HN008)	Unclassified	HN008	dsRNA	7596	38	250	3539	1179	128.0	KP861921
							3710	1236	140.0	
dsRNA virus 1 B275 (RsDSRV1/B275)	Unclassified	AG-1 IA B275	dsRNA1	2379	131	169	2078	692	78.7	JX976612
			dsRNA2	1811	115	298	1397	465	51.8	JX976613
putative virus 1 BR4 (RsV1/BR4)	Unclassified	AG-2.2 LP BR4	RNA	6311 partial	≥48	≥374	5887	1962	133.3	MK507780
M1 dsRNA	Unclassified	AG-3 Rhs 1A	dsRNA	6398 partial	≥9	419	639	212	24.0	AF020042
							5172	1723	196.5	
M2 dsRNA	Unclassified	AG-3 Rhs 1A	dsRNA	3570	421	884	2265	754	84.4	U51331

## Supplementary Materials

The following are available online at https://www.mdpi.com/1999-4915/11/12/1113/s1, Table S1: Additional mycoviruses reported to infect *R. solani*.
