# Supplementary material for: Viruses Infecting the Plant Pathogenic Fungus Rhizoctonia solani
Source: Viruses. 2019 Nov 30;11(12):1113. doi: 10.3390/v11121113 (PMC6950361; doi:10.3390/v11121113)
Supplement: Supplementary file 1 [file viruses-11-01113-s001.pdf]

Table S1: Additional partial mycoviruses reported to infect *R. solani* following NGS.

Name (abbr.)	Classification (family)	Segment(s)	Genome size (bp/nt)	Closest match (BLAST)	Similarity (%)	Accession Number
Rhizoctonia solani mitovirus 1	*Narnaviridae*	(+)ssRNA	2,462	Botrytis cinerea debilitation-related virus	35	KP900905
Rhizoctonia solani mitovirus 2	*Narnaviridae*	(+)ssRNA	2,865	Cryphonectria parasitica mitovirus 1	39	KP900911
Rhizoctonia solani mitovirus 3	*Narnaviridae*	(+)ssRNA	2,924	Tuber aestivum mitovirus	34	KP900912
Rhizoctonia solani mitovirus 4	*Narnaviridae*	(+)ssRNA	2,605	Botrytis cinerea debilitation-related virus	36	KP900913
Rhizoctonia solani mitovirus 5	*Narnaviridae*	(+)ssRNA	2,357	Tuber aestivum mitovirus 37	37	KP900914
Rhizoctonia solani mitovirus 6	*Narnaviridae*	(+)ssRNA	2,615	Ophiostoma mitovirus 6	37	KP900915
Rhizoctonia solani mitovirus 7	*Narnaviridae*	(+)ssRNA	3,085	Cryphonectria parasitica mitovirus 1	35	KP900916
Rhizoctonia solani mitovirus 8	*Narnaviridae*	(+)ssRNA	3225	Cryphonectria parasitica mitovirus 1	33	KP900917
Rhizoctonia solani mitovirus 9	*Narnaviridae*	(+)ssRNA	2,273	Clitocybe odora virus	37	KP900918
Rhizoctonia solani mitovirus 10	*Narnaviridae*	(+)ssRNA	2,701	Sclerotinia sclerotiorum mitovirus 1	33	KP900896
Rhizoctonia solani mitovirus 11	*Narnaviridae*	(+)ssRNA	3,283	Cryphonectria parasitica mitovirus 1	35	KP900906
Rhizoctonia solani mitovirus 12	*Narnaviridae*	(+)ssRNA	3,378	Clitocybe odora virus	31	KP900907
Rhizoctonia solani mitovirus 13	*Narnaviridae*	(+)ssRNA	3,039	Clitocybe odora virus	31	KP900908
Rhizoctonia solani mitovirus 14	*Narnaviridae*	(+)ssRNA	3,219	Clitocybe odora virus	46	KP900909
Rhizoctonia solani mitovirus 15	*Narnaviridae*	(+)ssRNA	3,901	Clitocybe odora virus	38	KP900910
Rhizoctonia solani mitovirus 9 DC17 (RsMV-9)	*Narnaviridae*	(+)ssRNA	672	Macrophomina phaseolina mitovirus 3	100	KX349058
Rhizoctonia solani mitovirus 16 (RsMV-16)	*Narnaviridae*	(+)ssRNA	687	Macrophomina phaseolina mitovirus 3	100	KX349057
Rhizoctonia solani mitovirus 17 (RsMV-17)	*Narnaviridae*	(+)ssRNA	663	Heterobasidion mitovirus 1	100	KX349059
Rhizoctonia solani mitovirus 18 (RsMV-18)	*Narnaviridae*	(+)ssRNA	633	Macrophomina phaseolina mitovirus 2	100	KX349060
Rhizoctonia solani mitovirus 19 (RsMV-19)	*Narnaviridae*	(+)ssRNA	675	Fusarium poae mitovirus 3	99	KX349056
Rhizoctonia solani mitovirus 20 (RsMV-20)	*Narnaviridae*	(+)ssRNA	978	Rhizoctonia solani dsRNA virus 1	82	KX349062
Rhizoctonia solani mitovirus 21 (RsMV-21)	*Narnaviridae*	(+)ssRNA	4100	Rhizoctonia solani mitovirus 13	51.13	MK372892
Rhizoctonia solani mitovirus 22 (RsMV-22)	*Narnaviridae*	(+)ssRNA	2177	dsRNA viral RdRp (mitochondrion) [Thanatephorus cucumeris]	80.15	MK490928
Rhizoctonia solani mitovirus 23 (RsMV-23)	*Narnaviridae*	(+)ssRNA	2792	Ceratobasidium mitovirus A	61.98	MK375261
Rhizoctonia solani mitovirus 24 (RsMV-24)	*Narnaviridae*	(+)ssRNA	2149	Macrophomina phaseolina mitovirus 2	85.48	MK372893
Rhizoctonia solani mitovirus 25 (RsMV-25)	*Narnaviridae*	(+)ssRNA	3767	Macrophomina phaseolina mitovirus 3	38.49	MK372894
Rhizoctonia solani mitovirus 26 (RsMV-26)	*Narnaviridae*	(+)ssRNA	2580	Rhizoctonia cerealis mitovirus	46.15	MK372895

Rhizoctonia solani mitovirus 27 (RsMV-27)	*Narnaviridae*	(+)ssRNA	3176	Rhizoctonia cerealis mitovirus	50.42	MK372896
Rhizoctonia solani mitovirus 28 (RsMV-28)	*Narnaviridae*	(+)ssRNA	2640	Rhizoctonia mitovirus 1	39.85	MK372897
Rhizoctonia solani mitovirus 29 (RsMV-29)	*Narnaviridae*	(+)ssRNA	2904	Binucleate Rhizoctonia mitovirus K1	42.14	MK372898
Rhizoctonia solani mitovirus 30 (RsMV-30)	*Narnaviridae*	(+)ssRNA	2760	Binucleate Rhizoctonia mitovirus K1	42.74	MK372899
Rhizoctonia solani mitovirus 31 (RsMV-31)	*Narnaviridae*	(+)ssRNA	3820	Rhizoctonia solani mitovirus 7	52.80	MK372900
Rhizoctonia solani mitovirus 32 (RsMV-32)	*Narnaviridae*	(+)ssRNA	3409	Rhizoctonia solani mitovirus 7	42.81	MK372901
Rhizoctonia solani mitovirus 33 (RsMV-33)	*Narnaviridae*	(+)ssRNA	2733	Rhizoctonia solani mitovirus 7	37.97	MK372902
Rhizoctonia solani mitovirus 34 (RsMV-34)	*Narnaviridae*	(+)ssRNA	3389	Rhizoctonia solani mitovirus 11	44.00	MK372903
Rhizoctonia solani mitovirus 35 (RsMV-35)	*Narnaviridae*	(+)ssRNA	3772	Rhizoctonia solani mitovirus 13	43.70	MK490929
Rhizoctonia solani mitovirus 36 (RsMV-36)	*Narnaviridae*	(+)ssRNA	2562	Rhizoctonia solani mitovirus 13	54.90	MK490930
Rhizoctonia solani mitovirus 37 (RsMV-37)	*Narnaviridae*	(+)ssRNA	3597	Rhizoctonia solani mitovirus 15	5.106	MK372904
Rhizoctonia solani mitovirus 38 (RsMV-38)	*Narnaviridae*	(+)ssRNA	3197	dsRNA viral RdRp (mitochondrion) [Thanatephorus cucumeris]	45.42	MK372905
ourmia-like virus 2 (RsOLV2)	*Botourmiaviridae*	(+)ssRNA	4104	Rhizoctonia solani ourmia-like virus 1 RNA 1	78.18	MK372906
ourmia-like virus 3 (RsOLV3)	*Botourmiaviridae*	(+)ssRNA	3223	Rhizoctonia solani ourmia-like virus 1 RNA 1	76.59	MK372907
ourmia-like virus 4 (RsOLV4)	*Botourmiaviridae*	(+)ssRNA	4557	Rhizoctonia solani ourmia-like virus 1 RNA 1	48.96	MK372908
ourmia-like virus 5 (RsOLV5)	*Botourmiaviridae*	(+)ssRNA	5234	Agaricus bisporus virus 15	25.89	MK372909
Rhizoctonia solani alphavirus-like 2 (RsALV2)	*Togaviridae*	(+)ssRNA	3396	Rhizoctonia solani RNA virus 1	69.38	MK507792
Rhizoctonia solani alphavirus-like 3 (RsALV3)	*Togaviridae*	(+)ssRNA	6752	Rhizoctonia solani RNA virus 3	83.12	MK507786
Rhizoctonia solani endornavirus 4 (RsEV4)	*Endornaviridae*	(+)ssRNA	20215	Endornavirus-like virus	35.16	MK393902
Rhizoctonia solani endornavirus 5 (RsEV5)	*Endornaviridae*	(+)ssRNA	16227	Rhizoctonia cerealis alphaendornavirus 1	47.31	MK393903
Rhizoctonia solani endornavirus 6 (RsEV6)	*Endornaviridae*	(+)ssRNA	15273	Morchella importuna endornavirus 2	45.18	MK393904
Rhizoctonia solani endornavirus 7	*Endornaviridae*	(+)ssRNA	14473	Rhizoctonia solani endornavirus 2	30.69	MK393905

(RsEV7)						
Rhizoctonia solani partitivirus 6 (RsPV6)	*Partitiviridae*	dsRNA	2401<br>2310	Fusarium poae partitivirus 2<br>Rosellinia necatrix partitivirus 8	50.96<br>58.08	MK507781<br>MK507782
Rhizoctonia solani partitivirus 7 (RsPV7)	*Partitiviridae*	dsRNA	1912<br>1867	Trichoderma atroviride partitivirus 1<br>Trichoderma atroviride partitivirus 1	68.08<br>31.69	MK507783<br>MK507784
Rhizoctonia solani partitivirus 8 (RsPV8)	*Partitiviridae*	dsRNA	1958<br>778	Rhizoctonia oryzae-sativae partitivirus 1<br>Heterobasidion partitivirus 20	53.19<br>42.35	MK532273<br>MK532274
Rhizoctonia solani dsRNA virus 6 (RsdsRNA6)	*Megabirnaviridae*	dsRNA	11847	Rhizoctonia fumigata mycovirus	30.95	MK507788
Rhizoctonia solani dsRNA virus 7 (RsdsRNA7)	*Megabirnaviridae*	dsRNA	3523	Rhizoctonia fumigata mycovirus	31.70	MK507789
Rhizoctonia solani dsRNA virus 8 (RsdsRNA8)	*Megabirnaviridae*	dsRNA	2905	Rhizoctonia fumigata mycovirus	30.83	MK507790
Rhizoctonia solani dsRNA virus 9 (RsdsRNA9)	*Megabirnaviridae*	dsRNA	2162	Rhizoctonia fumigata mycovirus	35.48	MK507791
Rhizoctonia solani dsRNA virus 10 (RsdsRNA10)	*Megabirnaviridae*	dsRNA	9416	Sclerotium rolfsii mycovirus dsRNA 1	37.37	MK532272
Rhizoctonia solani ourmia-like virus 2 (RsOLV2)	*Botourmiaviridae*	(+)ssRNA	4104	Rhizoctonia solani ourmia-like virus 1 RNA 1	78.18	MK372906
Rhizoctonia solani ourmia-like virus 3 (RsOLV3)	*Botourmiaviridae*	(+)ssRNA	3223	Rhizoctonia solani ourmia-like virus 1 RNA 1	76.59	MK372907
Rhizoctonia solani ourmia-like virus 4 (RsOLV4)	*Botourmiaviridae*	(+)ssRNA	4557	Rhizoctonia solani ourmia-like virus 1 RNA 1	48.96	MK372909
Rhizoctonia solani ourmia-like virus 5 (RsOLV5)	*Botourmiaviridae*	(+)ssRNA	5234	Agaricus bisporus virus 15	25.89	MK372908
Rhizoctonia solani fusarivirus 2 (RsFV2)	Unclassified	Unknown	10710	Rosellinia necatrix fusarivirus 2	40.57	MK558256
Rhizoctonia solani fusarivirus 3 (RsFV3)	Unclassified	Unknown	5959	Fusarium graminearum dsRNA mycovirus-1	38.09	MK558258
Rhizoctonia solani hypovirus 2 (RsFV2)	*Hypoviridae*	(+)ssRNA	9606	Sclerotium rolfsii hypovirus 1	27.16	MK558260
Rhizoctonia solani hypovirus 3 (RsFV3)	*Hypoviridae*	(+)ssRNA	5518	Agaricus bisporus virus 2	28.49	MK558255
Rhizoctonia solani putative virus 2 (RsPuV2)	Unclassified	Unknown	7137	Sanxia atyid shrimp virus 1	22.73	MK507785
Rhizoctonia solani putative virus 3 (RsPuV3)	Unclassified	Unknown	7214	Guarapuava tymovirus-like 1	31.91	MK532275
Rhizoctonia solani putative virus 4 (RsPuV4)	Unclassified	Unknown	7833	Gayfeather mild mottle virus	25.28	MK507793
